# Assessing the Accuracy of Spinal Instrumentation Using Augmented Reality (AR): A Systematic Review of the Literature and Meta-Analysis

**DOI:** 10.3390/jcm12216741

**Published:** 2023-10-25

**Authors:** Bhavya Pahwa, Tej D. Azad, Jiaqi Liu, Kathleen Ran, Connor J. Liu, Jovanna Tracz, Shahab Aldin Sattari, Jawad M. Khalifeh, Brendan F. Judy, Ali Bydon, Timothy F. Witham

**Affiliations:** 1 University College of Medical Sciences, GTB Hospital, New Delhi 110095, India; bhavyapahwa67@gmail.com; 2 Department of Neurosurgery, Johns Hopkins Hospital, Baltimore, MD 21287, USA; tazad1@jhmi.edu (T.D.A.); cliu185@jhmi.edu (C.J.L.); traczja@evms.edu (J.T.); ssattar3@jhmi.edu (S.A.S.); jkhalif1@jhmi.edu (J.M.K.); bjudy1@jhmi.edu (B.F.J.); abydon1@jh.edu (A.B.); 3 School of Medicine, Georgetown University, Washington, DC 20007, USA

**Keywords:** augmented reality, mixed reality, XVision, pedicle screw

## Abstract

Technological advancements, particularly in the realm of augmented reality (AR), may facilitate more accurate and precise pedicle screw placement. AR integrates virtual data into the operator’s real-world view, allowing for the visualization of patient-specific anatomy and navigated trajectories. We aimed to conduct a meta-analysis of the accuracy of pedicle screw placement using AR-based systems. A systematic review of the literature and meta-analysis was performed using the PubMed/MEDLINE database, including studies reporting the accuracy of pedicle screw placement using AR. In total, 8 studies with 163 patients and 1259 screws were included in the analysis. XVision (XVS) was the most commonly used AR system (595 screws) followed by the Allura AR surgical navigation system (ARSN) (462 screws). The overall accuracy was calculated as 97.2% (95% CI 96.2–98.1% *p* < 0.001). Subgroup analysis revealed that there was no statistically significant difference in the accuracy rates achieved by XVS and Allura ARSN (*p* = 0.092). AR enables reliable, accurate placement of spinal instrumentation. Future research efforts should focus on comparative studies, cost effectiveness, operative time, and radiation exposure.

## 1. Introduction

Pedicle screw placement is a fundamental spine surgery technique classically performed via an anatomy-based freehand technique or with fluoroscopic guidance [1]. However, screw misplacement is not uncommon and can have significant consequences [1]. Therefore, novel techniques and technologies have been developed to improve the accuracy and precision of pedicle screw placement. Augmented reality (AR) is an emerging technology that allows for virtual data to be integrated into the operator’s field of view in real time [2,3]. AR can provide the surgeon with real-time access to their patient’s three-dimensional (3D) anatomy without disrupting the surgical workflow [4]. AR was first used within spine surgery for pedicle screw placement but is now facilitating pelvic fixation, osteotomy planning, interbody placement, and tumor resection [5]. Recent studies have shown that AR-based systems can significantly enhance the accuracy of pedicle screw placement [6]. In this study, we aimed to perform a systematic review of the literature to assess the accuracy of AR-based systems for spinal pedicle screw placement. We also discuss the requirements, limitations, and barriers associated with such systems, providing insights that could assist in identifying gaps in the adoption of AR technology. 

## 2. Methods

### 2.1. Literature Search

A systematic search of the literature was performed following the Preferred Reporting Items for Systematic Reviews and Meta-Analyses (PRISMA) guidelines. The MEDLINE database was screened from inception until 31 May 2023, using the search syntax *(spine OR spinal) AND (“augmented reality” OR “mixed reality” OR “AR” OR Xvision OR HMD OR “head mounted” OR “head-mounted”) AND (screw OR pedicle OR fixation OR hardware)*.

### 2.2. Study Selection

Studies were reviewed after establishing the inclusion and exclusion criteria. We included the studies if they met the following criteria: involved ≥1 patient undergoing pedicle screw fixation using an AR-based system; provided accuracy data on screw placement; reported in English. Studies were excluded if they were conducted on cadavers or phantom models or if they were conference abstracts, reviews, technical notes, commentaries, or case reports. Articles were independently reviewed by the first two authors, using title/abstract screening followed by full-text evaluation. Any disagreements were resolved by the senior author. The reference lists of included studies were also manually screened to capture any additional studies not included by the initial search strategy.

### 2.3. Data Extraction

The required data were extracted by one author (B.P.) and then independently verified by an additional author (T.D.A.). The data included the patients’ demographics, comorbid medical conditions, number of screws, spinal level of screw placement, type of AR system, accuracy of screw placement, accuracy assessment scale, grade-wise accuracy, complications, and follow-up.

### 2.4. Evidence Assessment

The 2011 Oxford Center for Evidence-Based Medicine guidelines were utilized to assess the level of evidence of each study.

### 2.5. Statistical Analysis

The meta-analysis was conducted using MAJOR (Jamovi software, Version 2.4.11). A fixed-effects model was created to study the effect size of the screw accuracy achieved using AR. The fixed-effects model was chosen over the random-effect model because of the moderate heterogeneity detected using the I^2^ test (I^2^ = 31.87%). This could be attributed to the utilization of two different AR systems, the type of study design, and the involvement of a variety of spinal levels. A subgroup analysis was conducted to compare the accuracy of the Allura augmented-reality surgical navigation (ARSN) system with that of the XVision system (XVS) using the Student’s *t*-test; all statistics were two-tailed with a *p* < 0.05. To assess for publication bias, the Egger’s test was performed.

## 3. Results

### 3.1. Study Selection

**Figure 1:**
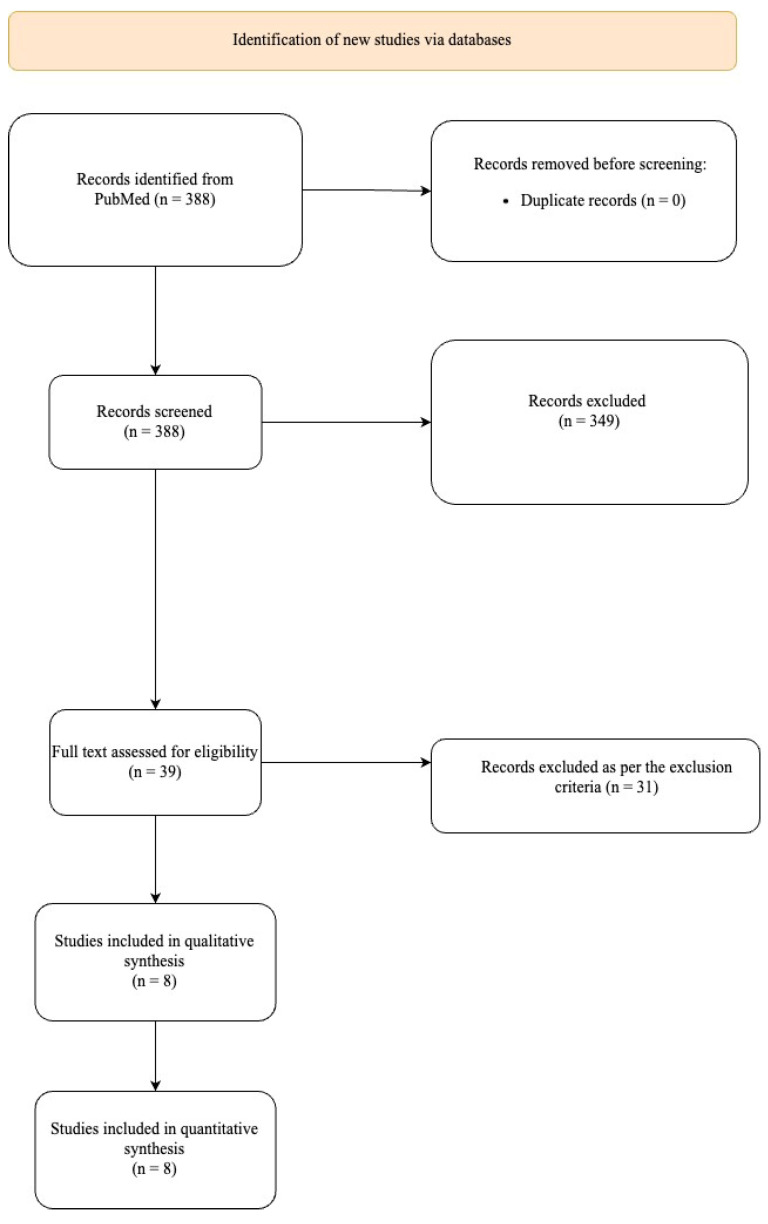
Detailed process of selection of studies according to PRISMA guidelines.

The initial search strategy returned a total of 388 potential studies for the meta-analysis (Figure 1). Following title/abstract screening, 349 studies were excluded, and the full texts of 39 studies were reviewed. After full-text review, 31 additional studies were excluded, resulting in 8 studies for quantitative meta-analysis (Table 1).

### 3.2. Patient Characteristics

A total of 1259 screws were placed in 163 patients (female, N = 94 (57.7%)) (Table 2).

Spinal deformity was the most common indication (N = 74, 45.4%) followed by degenerative spine disease (N = 35, 21.5%). The thoracic spine was the most frequent site of pedicle screw placement using AR (N = 431, 34.2%) followed by the lumbosacral spine (N = 221, 17.6%). One study did not mention the spinal level. The most commonly used AR system was the XVS (595 screws) followed by Allura ARSN (426 screws) and mixed reality (MR) (146 screws).

### 3.3. Accuracy

Most studies (N = 6, 75%) utilized the Gertzbein and Robbins (GR) classification to assess screw accuracy while two studies utilized the original Gertzbein classification. GR grading is a modification of the Gertzbein classification [14] as depicted in Table 3.

**Figure 2:**
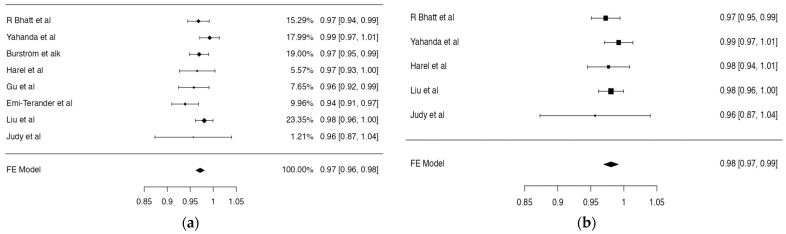
(**a**) Meta-analysis of the accuracies of spinal screw placement using augmented reality. (**b**) Meta-analysis of studies reporting the accuracy of the XVision system. Estimates for the main effect are provided by a shape and error bars, with estimates for summary effect provided by a diamond [6,8,9,10,11,12,13].

Six studies deconstructed their data to provide grade-specific accuracy (Table 1). Placement was termed “accurate” if the grading was below and inclusive of grade 1/B. Screw accuracy within individual studies ranged from 94% to 100%. The overall weighted accuracy was 97.2% (95% CI, 96.2–98.1% *p* < 0.001) (Figure 2a), meaning that, of 1259 screws, 1224 were accurately placed using AR (grade1/B or better) while 25 screws could not be accurately placed and were, therefore, placed freehand, guided either by fluoroscopy or other techniques, which were not explicitly reported by the studies. The effect size for accuracy achieved by the XVS was 98% (95% CI 97–99.2% *p* < 0.001) (Figure 2b). There was no statistically significant difference in the mean accuracy achieved by the XVS and the Allura ARSN (97.7 ± 1.6% vs. 95.5 ± 2.2%, *p* = 0.092).

### 3.4. Publication Bias Assessment

**Figure 3:**
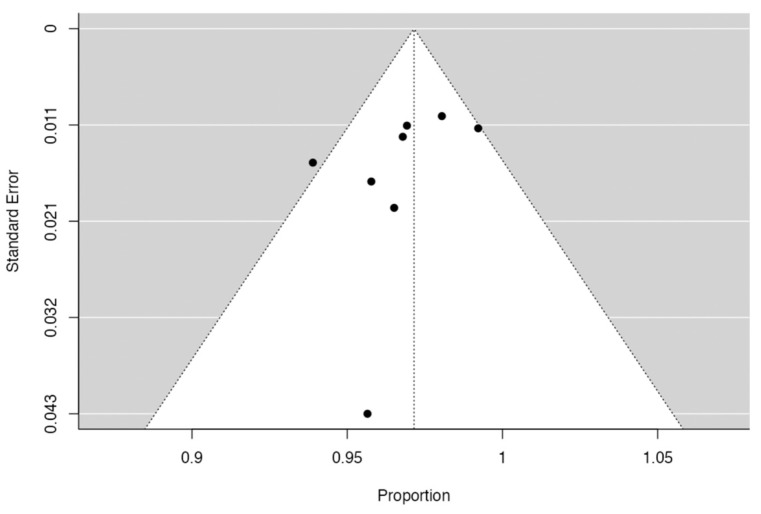
Funnel plot for the studies included in the analysis. Each dot represents and included study.

The *p* value of the Egger’s test was 0.131 (>0.05), suggesting low-to-minimal publication bias (Figure 3).

## 4. Discussion

Pedicle screw placement can be divided into two major schools of thought, namely freehand and imaging guided. Image guidance, classically, involves fluoroscopic guidance. Technological advancement has seen the emergence of new techniques for intraoperative navigation, i.e., robot assistance and AR [1]. Regardless of the technique utilized, an in-depth understanding and accurate identification of the anatomical landmarks remains critical for safe and precise screw insertion [6]. A systematic review reported an accuracy of 95.5% achieved by 3D fluoroscopy, demonstrating consistent success across all spinal levels. However, this technique often results in greater levels of radiation exposure [15]. Verma et al. found that the accuracy of computer-assisted screw placement (CASP) was significantly higher than that achieved freehand (93.3% vs. 84.7% *p* < 0.001) [16]. Additionally, the complication rate was 0% in patients undergoing CASP as compared to 2.3% for patients who underwent freehand screw placement [15]. However, the accuracy of CASP may depend on the angle between the camera and the surgical tool, the quality of the camera, the hand movement of the surgeon, the duration of surgery, and the environmental conditions. In an attempt to mitigate these challenges, robot-assisted screw placement (RASP) was introduced [9]. In our meta-analysis, an average weighted accuracy of 97.2% was achieved for patients undergoing pedicle screw placement using AR, comparable to rates cited in the most accurate freehand, CASP, and RASP studies [9,17]. These findings support the use of AR as a valuable tool for ensuring accurate and safe spinal instrumentation. The accuracy of individual studies varied from 94% (Emi-Terander et al. [6]) to 100% (Yahanda et al. [8]). Emi-Terander et al. intentionally chose larger screw diameters to maximize screw bony purchase and biomechanical stability. While this decision may have led to their higher incidence of cortical breaches, the authors posit that, without AR, it may not have been possible to place a screw at these levels due to miniscule pedicle diameters. The strength of AR lies in its potential to mitigate human limitations, such as mild hand tremors or trajectory variations. However, AR on its own might not entirely eliminate these challenges. Ultimately, the decision on surgical adjunct lies with the surgeon and the technology they are most comfortable with. AR and robot assistance both have pros and cons. While these adjuncts may have different ideal applications, currently, the decision on which adjunct to use should be made based on surgeon comfort. The recent literature, including the study by Vörös et al., has explored a mixed-model approach, combining the strengths of both AR and robotic systems [18].

Two AR systems have been utilized in spinal screw instrumentation, namely XVision AR-HMD and Allura ARSN. XVS is the only AR-HMD system approved by the Food and Drug Association (FDA), while Allura is currently only used as an off-label prototype [8]. XVS has a head-mounted display system that projects holographic images directly into the surgeon’s field of view, eliminating the need to look at a computer screen and facilitating an uninterrupted operative visual field. In contrast, the ARSN system features a C-arm panel with integrated video cameras and a monitor displaying AR-enhanced images. The most significant advantage of the ARSN system is its capacity to intraoperatively image misplaced screws via a built-in cone beam computed tomography device. In the context of thoracolumbar instrumentation, Burstrom et al. found comparable sensitivity and specificity in the identification of pedicle screw breaches using intraoperative CT compared to traditional postoperative CT imaging [10]. Intraoperative CT may minimize the need for returns to the operative room and postoperative follow-up imaging [10]. Two studies have described their human-trial results using ARSN in thoracolumbar spinal pedicle screws with a mean accuracy of 95.5% (94% Elmi-Terander et al. [8] and 97% Burstrom et al. [10]).

Comparatively, Gu and colleagues conducted a randomized prospective study to compare outcomes in patients undergoing lumbar pedicle screw insertion using mixed-reality (MR) versus freehand techniques. MR, a hybrid of augmented- and virtual-reality technologies, allows surgeons to superimpose virtual 3D holographic images onto the operative field. Gu and colleagues demonstrated a screw accuracy rate of 96% [7] when using MR, consistent with the accuracies observed in AR systems (Table 1) [19,20]. However, Gu et al. failed to mention the scale followed to calculate this accuracy rate, which needs to be mentioned to avoid confounded findings.

Navigated techniques appear to outperform freehand techniques in terms of operative time for actual screw placement. Butler et al. reported that the time taken to place each screw was 3 min 54 s using AR [21], which was less than that reported for fluoroscopy-guided (6.3 ± 3.0 min/screw) and robot-assisted screw placement (4.0 ± 1.1 min/screw) [22]. Khan et al. reported similar findings of 6.8 ± 0.9 min/screw for fluoroscopy-guided placement and 3.7 ± 1.8 min/screw for robotic placement [23]. However, these findings did not account for the setup time required for the robot or the AR system. The average total operative time required by the ARSN system was greater than that needed for fluoroscopy-guided placement, though statistically insignificant (403 min vs. 361 min, *p* =0.31) [8]. A similar effect was observed for deformity patients using this system [24]. It is likely that the entire operative team’s familiarity with the system and a standard imaging and registration process are necessary to achieve durable reductions in operative time when using AR.

Gu et al. reported a reduction in operative blood loss when using the MR system, when compared to the freehand group (MR, 382 cc; FH, 450 cc, *p* = 0.01) [7]. Similarly, intraoperative blood loss utilizing the ARSN system was nearly half of that lost during fluoroscopy-guided placement (628 ± 386 cc vs. 1165 ± 1103 cc, respectively *p* = 0.06) [8]. The ability of AR technology to potentially reduce operative time and blood loss suggests that AR may have the capacity to enhance intraoperative decision making and workflow.

Importantly, Boyaci et al. [25] sought to explore the use of AR technology for surgical education. The authors tested the ability of AR guidance on medical students and physicians (without formal surgical training) safely and accurately placing C2-3 transpedicular screws on cadaver models. Unsurprisingly, individuals in the AR group achieved a statistically higher Grade 0 safety ratio as compared to the freehand-alone group [25]. Thus, as AR technology becomes more accessible, surgical education will benefit from earlier hands-on experience as well as more standardized training.

While the potential benefits of AR technology in spinal instrumentation are significant, the associated capital costs are likely less than that of robotic systems [26,27]. These start-up costs most significantly impact on the technology’s expansion into lower-middle-income countries (LMICs) where AR systems could potentially provide the greatest long-term cost savings. Furthermore, considering that AR is a relatively new technology, it can be argued that outstanding accuracy outcomes may be predominately achieved in the hands of the highly experienced surgeons, as exemplified by the 100% accuracy rate by Yahanda et al. [6] The correlation between accuracy and the surgeon’s experience is directly proportional, as revealed by Rivkin et al., who noted an accuracy increase from 86.8% for 30 cases to 98.9% for 270 cases [28].

### Limitations

Factors such as obesity, age, and osteoporosis are pivotal in such studies since they can affect screw breach [29,30]. However, our meta-analysis aimed to consolidate the findings of individual studies, and as such, we relied on the data provided in those studies. Many of the original articles with relatively small cohorts did not provide detailed patient demographics or did not statistically analyze these potential risk factors. Incorporating a new statistical analysis based on these factors would require access to raw, patient-level data, which is unfortunately beyond the scope of our current meta-analysis. Additionally, limited follow-up was reported for the included studies. Only one study reported a short-term follow-up at two weeks postoperation. Future studies would benefit from extended follow-up periods to provide a more comprehensive understanding of AR applications. Furthermore, only two studies reported the operative duration as a variable. While AR navigation requires additional steps for setup including intraoperative imaging and navigation array registration, it may expedite intraoperative decision making and enable faster instrumentation placement. Future studies should evaluate the overall effect of AR navigation on total operative time, as well as time specifically for hardware placement. Another limitation was the inability to further stratify results based on the anatomical risk associated with pedicle screw placement. Specifically, screws placed in high-risk areas could present different challenges. Future studies with a more detailed dataset would be beneficial in shedding light on this crucial aspect.

## 5. Conclusions

Augmented reality (AR) is an emerging technology with promising results in spinal surgery. The early data indicate that AR navigation for pedicle screw placement is safe and accurate. Future investigations into long-term patient outcomes the cost effectiveness of AR navigation will be important to help determine the best role for this technology in the future of spinal surgery.

## Figures and Tables

**Table 1:** Summary findings of studies included in meta-analysis.

Author, Year	Study	Technology	No. of Screws	No. of Patients	Gender	Mean Age (yrs)	Spinal Level	Pathology	Accuracy Assessment Tool	Accuracy (%)	Breach Grade
Gu et al., 2020 [7]	RCT	MR	142	25	11 M 14 F	44.32	142 L	Lumbar spondylolisthesis (n = 7)	NR	95.77	NR
Emi-Terander et al., 2020 [8]	Prospective	Allura ARSN	262	20	9 M 11 F	30	166 T, 96LS	Scoliosis (n = 13), kyphosis (n = 2), other (n = 5)	G	93.9	63.4% Grade 0 30.5% Grade 1 6.1% Grade 2
Liu et al., 2021 [9]	Retrospective	XVS	205	28	11 M 17 F	62.5 *	67 T, 112 L, 26 S1	12 degenerative disease; 12 deformity correction; 3 tumor; and 1 trauma	GR scale	98	94% Grade A 4% Grade B 1.5% Grade C 0.5% Grade D
Yahanda et al., 2021 [6]	Retrospective	XVS	63	9	5 M 4 F	71.9	32 T, 31 L	4 tumors, 3 degenerative disease, 1 spinal deformity, and 1 infection	GR scale	100	96.8% Grade A, 3.2% Grade B
Burström et al., 2021 [10]	Retrospective	Allura ARSN	260	20	9 M 11 F	30.5	166 T, 94 LS	13 scoliosis, 2 kyphosis, 3 lumbar spondylolisthesis, 1 lumbar spinal stenosis, 1 lumbar degenerative disk disease	GR scale	97	NR
R Bhatt et al., 2022 [11]	Prospective	XVS	218	32	13 M 19 F	50.9	TLSP	6 deformity, 5 instability, 9 postlaminectomy syndrome, 2 pseudoarthrosis, 10 stenosis	GR scale	97.1	91.8% Grade A, 5.3% Grade B
Harel et al., 2022 [12]	Prospective	XVS	86	17	7 M 10 F	60.23	40 L, 46 LS	17 spondylosis	GR scale	97.7	84.9% Grade A, 12.8% Grade B, 2.3% Grade C
Judy et al., 2023 [13]	Retrospective	XVS	23	12	4 M 8 F	63 *	S2 alar-iliac	3 degenerative disease, 8 deformity, 1 tumor	G	95.6	91.3% Grade 0, 4.3% Grade 1, 4.3% Grade 3

* Median age. Abbreviations: MR, mixed reality; XVS, XVision system; ARSN, augmented-reality navigation system; G, Gertzbein classification; GR, Gertzbein and Robbins grading; T, thoracic; L, lumbar; S, sacral; LS, lumbosacral; NR, not reported; P, pelvic.

**Table 2:** Patient characteristics.

Parameter	Frequency
Number of Screws	1259
Thoracic	431 (34.2%)
Lumbar	194 (15.4%)
Lumbosacral	221 (17.6%)
S1	26 (2.1%)
S2 alar-iliac	23 (1.8%)
No. of Patients	163
Males	69 (42.3%)
Females	94 (57.7%)
Conditions	
Deformity	74 (45.4%)
Tumor	8 (5%)
Degenerative disease	35 (21.5%)
Spondylolisthesis	10 (6.1%)
Trauma	1 (0.6%)
Infection	1 (0.6%)
Pseudoarthrosis	2 (1.2%)
Postlaminectomy syndrome	9 (5.5%)
Others	5 (3.1%)
AR System	
XVision, head mounted	595 screws
Allura ARSN	462 screws
Mixed reality	142 screws

**Table 3:** Relation between Gertzbein classification and Gertzbein and Robbins grading of accuracy for pedicle screw placement.

Gertzbein Classification	Gertzbein and Robbins Grading	Interpretation
Grade 0	Grade A	No cortical breach or screw within pedicle
Grade 1	Grade B	0–2 mm breach
Grade 2	Grade C	2–4 mm breach
Grade 3	Grade D	4–6 mm breach
	Grade E	>6 mm breach

## Data Availability

Not applicable.
